# Correspondence on “DC Electric Fields Promote Biodegradation of Waterborne Naphthalene in Biofilter Systems”

**DOI:** 10.1021/acs.est.5c06074

**Published:** 2025-10-01

**Authors:** Frank-Dieter Kopinke, Stefano Salvestrini

**Affiliations:** † Faculty of Chemistry and Mineralogy, Leipzig University, Johannisallee 29, Leipzig D-04103, Germany; ‡ Department of Environmental, Biological and Pharmaceutical Sciences and Technologies, University of Campania “Luigi Vanvitelli”, via Vivaldi 43, 81100 Caserta, Italy

Wick et al.^1^ have investigated the biodegradation of naphthalene (NAH) in aqueous solution in a packed-bed flow-through reactor, specifically the effect of direct current (DC) electric fields on degradation rates. They observed moderately positive DC effects. They interpret them as effects of electro-osmotic flow (EOF) on mass-transfer rates.

Our criticisms are that (i) the applied kinetic model is inappropriate for the applied experimental setup, (ii) the numerical data treatment is erroneous, and (iii) the mechanistic interpretation based on the EOF is hardly plausible. In addition, some relevant electrochemical effects are disregarded. We substantiate our critical evaluation step by step as follows.

The authors^1^ use *q*_transfer_ *= k*(*C*_0_ – *C*_cell_) (eq 2 in ref 1) to describe substrate-transfer rate *q*_transfer_ from the water bulk phase to the cell surface. It is the product of mass-transfer rate coefficient *k* (in L g_protein_^–1^ min^–1^) and concentration gradient Δ*C*_diffusion_ (in mol L^–1^) as the driving force of diffusion. Usually, Δ*C*_diffusion_ is specified as *C*_bulk_ – *C*_surface_. In this study, bulk-phase concentration *C*_bulk_ is substituted by initial substrate concentration *C*_0_. This substitution leads to a systematic overestimation of Δ*C*_diffusion_ and consequently to an underestimation of calculated mass-transfer coefficients *k*. The extent of this underestimation depends on the (unknown) substrate concentration profile along the reactor length. An illustration of this bias is presented in the Supporting Information. Note that evaluation of the derived *k* values is the basis of “quantifying the effect of the EOF on the biodegradation”, as claimed in ref 1.

The applied mass-transfer model, the film or boundary layer model,^2^ is usually applied for well-mixed fluid phases in contact with solid surfaces, typically achieved in batch experiments. In the present study, the water flows laminarily through the particle bed rather than being well mixed on the microscale. It has large radial and axial concentration gradients. The applied kinetic model is *a priori* inappropriate for the used reactor type, rather than being covered by a “simplification” approach. A similar mismatch between experimental setup and applied kinetic model has been addressed recently.^3^

In the next step, we examine the numerical correctness of the presented data. Unfortunately, the authors did not explicitly present mass-transfer coefficients *k*. Instead, they present bioavailability numbers *B*_n_, which are connected with mass-transfer coefficients by the equation *B*_n_ = *k*/(*q*_max_/*K*_s_) (eq 4 in ref 1), where the quotient *q*_max_/*K*_s_ is called the microbial specific activity of the bacteria. Having assumed steady state conditions (i.e., *q*_transfer_ = *q*_cell_), Wick et al.^1^ used the Best equation (eq 3 in ref 1) to calculate *k* values. The extraction of *k* from this equation seems to us to be unnecessarily tortuous because it cannot be explicitly resolved for *k*. We prefer a more direct way simply by combining eqs 1 and 2 in ref 1. This results in

i $$k=q_{transfer}/\left(C_{bulk}-C_{cell}\right)=q_{cell}/\left(C_{bulk}-C_{cell}\right)=q_{cell}/\left[C_{bulk}-K_{s}/\left(q_{\max}/q_{cell}-1\right)\right]$$

This equation contains only measured data from the column experiments (*q*_cell_ and *C*_bulk_ ≈ *C*_0_ for low NAH conversion) and from batch experiments (*q*_max_ and *K*_s_). We performed a simple analysis of the data sets presented in Wick et al.^1^ and came to a surprising result. All directly calculated *k* values and the corresponding *B*_n_ values are significantly different from those presented in ref 1. All *B*_n_ values derived from directly calculated *k* values are a factor of 30 ± 20 smaller than those presented in Figure 3 and discussed extensively throughout ref 1. An example of the numerical calculations is presented in the Supporting Information. When the set of key parameters, *B*_n_ and *k* values, derived by Wick et al.^1^ is erroneous by 1–2 orders of magnitude, it does hardly make sense to follow their discussion in detail. We demonstrate in the Supporting Information how erroneous bioavailability numbers *B*_n_ lead to wrong conclusions about rate-controlling steps.

Nevertheless, it is reasonable to reconsider the mechanistic view of this study on a qualitative basis, i.e., how the EOF can accelerate the mass transfer from a laminary flowing bulk phase to a nonporous particle surface. The EOF is a surface-associated water movement taking place at a nanometer distance above the particle surface.^4,5^ Its velocity (about 3 × 10^–7^ m s^–1^) is 2–3 orders of magnitude lower than the Darcy velocity of the bulk water flow (about 10^–4^ m s^–1^). Wick et al.^1^ hypothesize that the EOF reduces the “effective thickness” of the diffusive boundary layer on the surface of the glass beads, respective the cell surface. The thickness of this boundary layer is usually on the scale of micrometers. From the correctly calculated mass-transfer coefficients *k* ∼ *D*_NAH_/δ_layer_ (with *D*_NAH_ being the diffusion coefficient of NAH in water), we estimate an effective layer thickness δ_layer_ ≈ 10^2^ μm (calculation not outlined here). It is not plausible for us how a very thin, very slow EOF can accelerate the molecular diffusion of the substrate across a much thicker, much faster moving laminar water layer. We challenge the plausibility of this mechanism, which comes from mass transfer in capillaries or inside porous particles,^4,5^ where EOF does not interact with much stronger advective flows.

Another issue worth considering is the “electrical part” of this study. The authors mention a steady current intensity of about 3.6 mA but do not consider possible biochemical consequences of this current. The water inflow passes first the cathode at the top of the reactor column (Figure S1). Therefore, the redox milieu inside the bioreactor may be affected by reduction equivalents, e.g., oxygen depletion. A 3.6 mA electrical current corresponds to a flux of 37 nmol s^–1^ electron equivalents or to a concentration of 2–12 mM of reduction equivalents in the inlet water flow (3.1–16.5 μL s^–1^). These concentrations are much higher than the applied substrate concentrations (0.027–0.078 mM NAH). Disregarding redox conditions and dissolved oxygen concentrations seems to us risky for a quantitative interpretation of biodegradation processes. A critical consideration of electrochemical aspects of the applied setup is presented in the Supporting Information.

In summary, Wick et al.^1^ investigated DC field effects on biodegradation rates in a fixed-bed reactor. They interpret them in terms of the EOF effects on mass-transfer rates. Our criticisms are that (i) the presented data treatment is erroneous, (ii) the applied kinetic model is inappropriate, and (iii) the mechanistic conclusions are not covered by the experimental data and are not in harmony with established mass-transfer rules. This mismatch should not remain undisputed in *Environmental Science & Technology*.

## Supplementary Material



## Notes

Illustration of various concentration gradients in the flow-through reactor column, example of correct versus erroneous calculation of mass-transfer coefficients, calculation of substrate cell surface concentrations, identification of rate-controlling steps, understanding the dependence of specific degradation rates on water flow velocities, and adverse effects of electric current on the redox milieu in the bioreactor (PDF)
