# Supplementary material for: Correspondence on “DC Electric Fields Promote Biodegradation of Waterborne Naphthalene in Biofilter Systems”
Source: Environ Sci Technol. 2025 Oct 1;59(40):21759–60. doi: 10.1021/acs.est.5c06074 (PMC12529951; doi:10.1021/acs.est.5c06074)
Supplement: Supplementary file 1 [file es5c06074_si_001.pdf]

Supporting Information, for Reviewers only, to the manuscript: Correspondence on "DC Electric Fields Promote Biodegradation of Waterborne Naphthalene in Biofilter Systems"

Frank-Dieter Kopinke and Stefano Salvestrini

1. Illustration of the different concentration gradients ΔC_{film} as driving force for the diffusive mass transfer inside a fixed-bed flow-through reactor.

$\Delta C_{film} = C_{bulk} - C_{cell}$ (i.e., the correct gradient) vs. $\Delta C_{ref[1]} = C_0 - C_{cell}$ (used in Wick et al.[1])

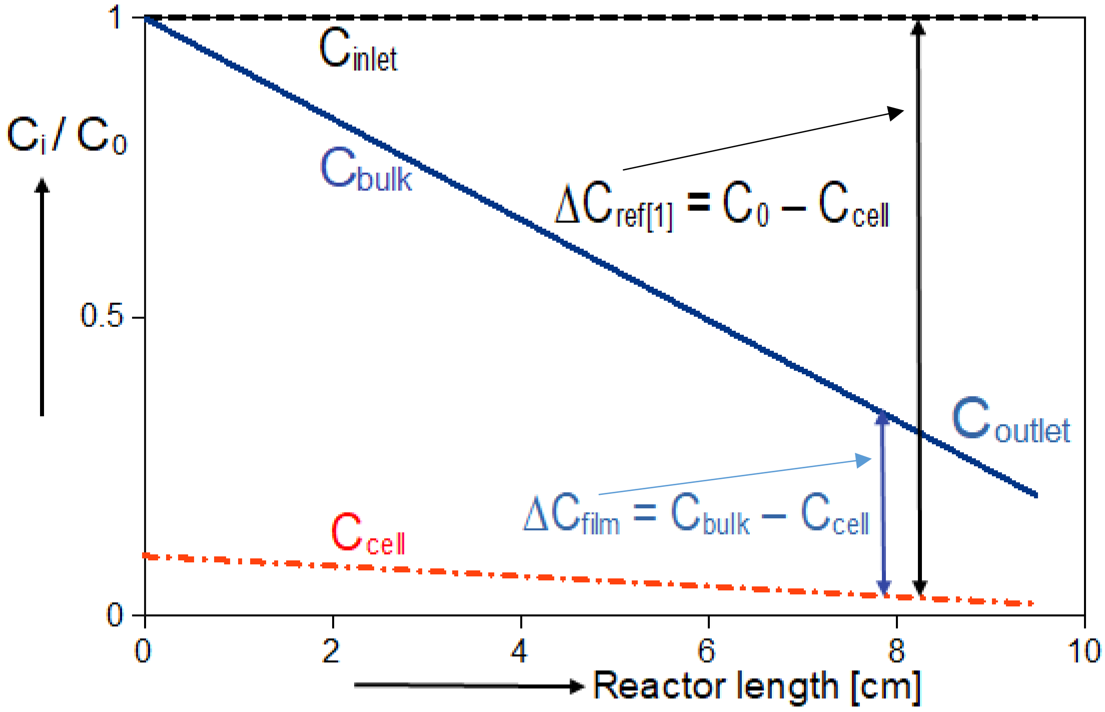

The diagram (adapted from data in [1]) shows three substrate concentration profiles in the fixed-bed reactor along its length axis (0-10 cm). The inlet concentration C_{inlet} (= C_0) is a constant (dashed black line). The bulk-phase concentration C_{bulk} (blue line) decreases along the reactor axis. In the present case, we approximate it as a straight line, i.e., it reflects a pseudo-zero-order reaction kinetics with a final substrate conversion of about 80%. The reaction order is tentatively assumed but not essential. C_{cell} (broken red line) is approximated from the experimental data (see below). Note, C_{cell} is low compared to C_{bulk}.

Wick et al. (subscript ref.[1] in the above diagram) used $\Delta C_{ref[1]} = C_0 - C_{cell}$, indicated by the black double-arrow. The true concentration gradient is $\Delta C_{film} = C_{bulk} - C_{cell}$, indicated by the blue double-arrow.

It is obvious that the approximation of Wick et al.[1] overestimates systematically the active concentration gradient. This overestimation is the higher the larger the substrate depletion is,

i.e., with increasing length coordinate and with decreasing outlet concentration. The overestimation of ΔC_{film} causes an underestimation of the corresponding mass-transfer coefficient $k = q_{cell}/\Delta C_{film}$.

A reduction of the bias could be achieved by using an averaging of $C_{bulk} = 0.5\cdot(C_{inlet} + C_{outlet})$. Wick et al.[1] did not mention or made use of this approximation.

Moreover, we see a basic problem in the definition of a (uniform) bulk-phase concentration C_{bulk} on the micro-scale in case of a laminar (laminated) water flow with strong substrate depletion at the reactive surfaces.

Mass-transfer kinetics in laminar flows is well described in the pertinent literature (see e.g. [2]). The concentration profile inside a laminar flow is quantitatively described by means of the complementary error function (erfc) with an argument wherein the maximum flow velocity v_{max} and the molecular diffusion coefficient $D_{substrate}$ are key parameters. All rate laws have to be integrated along the reactor axis due to the changing concentrations. None of these methods was applied in Wick et al.[1].

2. Example for numerical calculation of mass-transfer coefficients k and bioavailability numbers B_n with a data set from Figures 2 and 3 from Wick et al.[1]

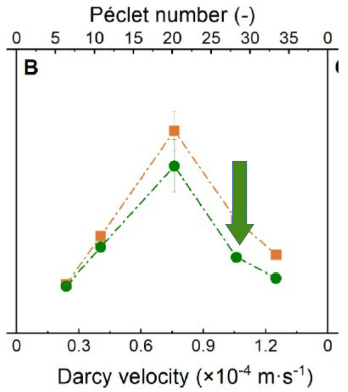

We take one (arbitrarily selected) data point from Figure 2 (green arrow in the clipping), namely the 4th green point in Figures 2B and 3B (without DC field) with the following data set:

Initial NAH concentration $\boldsymbol{C_0}$ **= 51 µM**

$C_{out}/C_{in} \approx 0.85$ (i.e. 15% NAH conversion, from Figure 1-I in [1])

Darcy velocity $U = 1.1\cdot10^{-4}$ m s^{-1}

Degradation rate $\boldsymbol{q_c}$ **≈ 150 nmol mg$_{\text{protein}}^{-1}$ min^{-1}** (from Figure 2, q_c in [1] is q_{cell} in our Comment) and Bioavailability number $\boldsymbol{B_n}$ **≈ 0.55** (from Figure 3). Both numbers are taken visually from the presented diagrams.

$\boldsymbol{K_s}$ ($K_s = K_m$ in eq. 3) **= 2.5 µM** and $\boldsymbol{q_{max}}$ **= 555 nmol mg$_{\text{protein}}^{-1}$ min^{-1}** are taken from the text (see "NAH Biodegradation in the Absence of DC" subsection in the RESULTS section). They are fitting parameters, according to eq. 1, from batch experiments with suspended cells. Unfortunately, the authors do not present any experimental data from their batch experiments nor error ranges of the derived fitting parameters. Therefore, the reader has to rely on the

characteristic cell parameters K_s and q_{max} 'blindly'. We adopt here the unit "$mg_{protein}$" used in [1] although, according to the IUPAC-rules, subscripts on units are not correct.

The selected example with a low NAH conversion degree (≈15%) is flawed by a relatively low model bias due to the author's approximation $C_{bulk} = C_0$. Hence, the numerical discrepancies in parameter values (k and B_n) are not due to an inadequate model structure but to an erroneous data treatment of the authors, in our view.

We calculate the mass-transfer coefficient k according to the direct method, i.e., with our eq. *i*, which is obtained by combining equations 1 and 2 in [1]. All input data for using this equation are written above in bold format.

$$k = q_{transfer}/(C_{bulk} - C_{cell}) = q_{cell}/(C_{bulk} - C_{cell}) = q_{cell}/[C_{bulk} - K_s/(q_{max}/q_{cell} - 1)] \qquad \text{(eq. } i\text{)}$$

For the indicated data point, it results $k = 0.003$ L mg^{-1} min^{-1} and $B_n = k{\cdot}K_s/q_{max} = 0.0135$. The latter value has to be compared with $B_n \approx 0.55$ from Figure 3B in [1]. The discrepancy is a factor of 41, in this case.

Next, we check the validity of the two k-values, $k = 0.003$ L $mg_{protein}^{-1}$ min^{-1} (from our direct method) and $k = 0.55{\cdot}q_{max}/K_s = 0.12$ L $mg_{protein}^{-1}$ min^{-1} (from [1]) when implementing them into the Best-equation. We acknowledge that Wick et al.[1] revised the erroneous version of the Best-equation cited from Harms and Bosma[3] (ref. [42] in [1]).

Best-equation (eq. 3 in [1])

$$q_c = q_{max}\frac{C_0 + K_s + q_{max}k^{-1}}{2q_{max}k^{-1}}\left[1 - \sqrt[2]{1 - \frac{4C_0q_{max}k^{-1}}{(C_0 + K_m + q_{max}k^{-1})^2}}\right]$$

It results $q_{cell} = 150$ and 527 nmol $mg_{protein}^{-1}$ min^{-1}. The first value (this comment) matches perfectly the experimental value. The second value (from Wick et al.[1]) overestimates the NAH mass flux significantly: $q_{cell} = 527$ nmol $mg_{protein}^{-1}$ min^{-1} is close to the maximum achievable cell flux of $q_{max} = 555$ nmol $mg_{protein}^{-1}$ min^{-1}, which is the upper limit of the model. The comparison shows that the Best-equation allows to calculate correct mass fluxes, provided correct mass-transfer coefficients k are used. The k-values in Wick et al.[1] do not fit the measured NAH-transformation rates.

3. It is also useful to **calculate the NAH concentration at the cell surface C_{cell}** from eq. *i*:

$$C_{cell} = K_s \cdot \{q_{cell}/q_{max} /(1 - q_{cell}/q_{max})\} = 2.5\ \mu M \cdot \{150/555/(1 - 150/555)\} = 0.93\ \mu M$$

This means, the mean NAH bulk-phase concentration of 51 μM (column inlet) to 43 μM (column outlet, $C_{outlet} = 0.85 \cdot C_0$) is depleted down to about 1 μM near the cell surface, due to biochemical degradation and molecular diffusion across the laminar water body. Obviously, the NAH conversion rate is mainly controlled by mass-transfer processes rather than its microbial specific activity (q_{max}/K_s).

Wick et al.[1] discussed different scenarios depending on the bioavailability numbers B_n. The overall transformation rate is assumed to be mass-transfer controlled when $B_n < 1$ and to be controlled by the biotransformation rate when $B_n > 1$. Although we do not agree with this perspective, it is instructive to reconsider this discussion on the basis of correct bioavailability numbers. We look at the two maximum data points in Figure 2B, with $q_{cell} \approx 350$ and 410 nmol $mg_{protein}^{-1}$ min^{-1} without and with DC field, respectively. The corresponding B_n-values in Figure 3B are $B_n \approx 1.2$ and 2.0. This means, according to Wick et al.[1] these overall transformation rates are biochemically controlled (criterion: $B_n > 1$). When using the correct k-values, it results $B_n = 0.034$ and 0.042. These values reflect a mass-transfer controlled regime (criterion: $B_n < 1$). This is the opposite of Wick et al.'s[1] conclusion.

We elaborate this example in such detail in order to demonstrate the significant adverse implications of the erroneous data treatment in [1]. It is more than playing with numbers, it leads to significant mechanistic misinterpretation.

4. Finally, we want to address another remarkable finding in [1]: Figure 2 shows **the dependence of specific NAH degradation rates** q_{cell} (in nmol $mg_{protein}^{-1}$ min^{-1}) **on water flow velocities** U (in 10^{-4} m s^{-1}): $q_{cell} = f(U)$. All these profiles, independent of DC application or initial NAH concentration, show pronounced maxima at mean flow velocities. When increasing the flow velocity moderately from $0.76 \cdot 10^{-4}$ m s^{-1} to $1.1 \cdot 10^{-4}$ m s^{-1} the specific degradation rates decrease by more than 50%. Note that these rates q_{cell} are not normalized to the NAH conversion extent ($1 - C_{out}/C_{in}$) but rather to the amount of immobilized biomass in the column (per $mg_{protein}$). These are experimental data. They are not affected by model assumptions and erroneous calculations. Therefore, they have to be accepted as such. They are, however, counterintuitive to the experienced reader. Usually, a mass-transfer controlled process is to be expected more effective at higher flow rates. The experimental data show the opposite tendency. We cite one of several explanation hypotheses of the authors: „While

biodegradation rates increased at increasing Darcy velocity up to $U > 0.8 \cdot 10^{-4}$ m s^{-1}, they again decreased at higher flow rates. Such an observation is in line with the literature describing contaminant biodegradation in porous media at comparable flow conditions under both growth and nongrowth conditions and was explained by the dependency of biodegradation rates on the mean contact times (i.e., hydraulic retention time) between a contaminant and bacteria due to microbial substrate uptake restrictions.[53–56]" It is plausible that NAH conversion rates decrease with decreasing residence time inside the bioreactor, when (i) considering *rates* as extents of NAH conversion = $1 - C_{out}/C_{in}$. But it is not plausible, when (ii) considering *rates* as amounts of converted substrate (NAH) per unit biomass and per time interval = $n_{NAH\_converted}/m_{cells\_immobilized}/\Delta t = v_{water} \cdot (C_{in} - C_{out})/m_{cells\_immobilized}$, with v_{water} (µL s^{-1}) as water flow. The latter (ii) is given in Figure 2, a biomass (as $mg_{protein}$) normalized conversion rate in units of $nmol_{NAH}$ $mg_{protein}^{-1}$ min^{-1}. Possibly, the authors have confused two different types of conversion rates in their explanation. We also inspected carefully the four references [53-56] given as support from the literature for the surprising findings in [1]. None of them is applicable, in our view, to the discussed experiments of Wick et al.[1]. As an example, we cite a passage from reference [56]: „While the total amount of degraded toluene increased with the flow velocity, toluene removal efficiency, i.e., the percentage of the injected toluene mass which was degraded while being transported through the column, decreased." A perfunctory interpretation could see a support of Wick et al.'s[1] findings in this statement. Actually, Grosbacher et al.[56] described biodegradation rates of type (i), i.e., biodegradation efficiencies rather than type (ii) rates. Type (ii) rates are not considered at all in [56].

Wick et al.[1] used *k*-values normalized to the amount of proteins in cells, in L $mg_{protein}^{-1}$ min^{-1}. One can transfer these units in a more illustrative unit, m s^{-1}, according to $k' = D/\delta$ with D as molecular diffusion coefficient (in m^2 s^{-1}) of the substrate in water and δ as the effective thickness (in m) of the stagnant boundary layer. We performed this transformation (with $D_{NAH} \approx 10^{-9}$ m^2 s^{-1}). It yields $\delta = D/k' \approx 2$ µm$/B_n$ (derivation not outlined herein). The B_n-values from 0.1 to 4 presented in Figure 3 in [1] yield $\delta \approx$ 10-20 µm (at U_{Darcy} = 0.2 and 1.2 m s^{-1}) to 0.5 µm (at U_{Darcy} = 0.8 m s^{-1}), i.e., at the maxima of the B_n-profiles. 0.5 µm are an extremely thin boundary layer, which can hardly be explained by hydrodynamic conditions.[2] In summary, two outcomes of the mass-transfer model calculations are hardly plausible: the profiles $k = f(U_{Darcy})$ and the thickness of the boundary layer $\delta \leq 1$ µm under laminar flow conditions.

Provided we rely on the primary experimental data of the study, i.e., the NAH degradation rates in Figures 1 and 2, the following question arises: What might be the reason

of the addressed issues? In our view, it is the inadequateness of the applied kinetic model, which assigns all observed effects, inclusive the observed DC-field effects, to mass-transfer effects, i.e., finally to effects of EOF. We think this narrative is not covered by the presented data, in particular not supported by the applied kinetic model.

5. Another issue worth to be considered in some detail is the '**electrical part**' of the study.

(i) The DC-field strength E (in V cm^{-1}), a key-parameter for treatment of the electroosmotic flow (EOF), is calculated in [1] as the voltage difference between cathode and anode (Figure S1), i.e., E = 5 V/10 cm = 0.5 V cm^{-1}. Apparently, the potential drop near the electrodes was not taken into account. However, it does not contribute to the average field strength inside the fixed bed. Hence, E-values are overestimated in [1] to an undefined extent.

(ii) More importantly, the authors give a steady current intensity of about 3.6 mA under flow conditions (3 to 16.5 µL s^{-1} flow of buffer solution) in their experiments, but do not consider chemical or biochemical effects of this current. The water inflow passes first the cathode at the top of the column (Figure S1). Therefore, the redox milieu inside the bioreactor may be affected by reduction equivalents. We do not know what are the predominant cathodic reactions ($O_2 \rightarrow H_2O_2$ or $H_2O \rightarrow H_2$ or others). 3.6 mA electrical current correspond to 36 nmol s^{-1} electron equivalents or 1 to 6 mM of 'dissolved reduction equivalents'. These concentrations must be related to 0.027 to 0.078 mM substrate (NAH) concentration and about 0.7 mM dissolved O_2 concentration (in air saturated solution) in the column inflow. Hence, the amount of reduction equivalents released from the cathode under DC conditions is in the order of other reactant concentrations or even much higher. The biodegradation of NAH is most likely an aerobic process wherein the available oxygen concentration can be expected to play a significant role. Even in case of a dominant cathodic water electrolysis (H_2 formation rather than O_2 reduction), dissolved O_2 would be significantly depleted by physical stripping by evolving hydrogen gas. Neither an oxygen depletion nor a possible H_2O_2 formation nor any biochemical effects of dissolved H_2 have been considered in [1]. The blank experiments presented in Figure S4 with heat-inactivated microorganisms (no significant NAH conversion) do not help here, because they do not tell anything about possible redox effects with *active* microorganisms. Disregard of oxygen concentration seems to us risky for solid interpretation of aerobic biodegradation processes.

References

(1) He, J.; Castilla-Alcantara, J. C.; Ortega-Calvo, J. J.; Harms, H.; Wick, L. Y. DC Electric Fields Promote Biodegradation of Waterborne Naphthalene in Biofilter Systems. *Environ. Sci. Technol.* **2024**, *58*, 18234−18243.

(2) https://faculty.ksu.edu.sa/sites/default/files/che318_part4_convective-mass-transfer-rev2a.pdf.

(3) Harms, H.; Bosma, T. N. P. Mass Transfer Limitation of Microbial Growth and Pollutant Degradation. *J. Ind. Microbiol. Biotechnol.* **1997**, *18*, 97−105.

(56) in [1] Grosbacher, M.; Eckert, D.; Cirpka, O. A.; Griebler, C. Contaminant Concentration Versus Flow Velocity: Drivers of Biodegradation and Microbial Growth in Groundwater Model Systems. *Biodegradation* **2018**, *29*, 211−232.
